# Editorial: Diabetes and depression

**DOI:** 10.3389/fcdhc.2023.1221640

**Published:** 2023-06-23

**Authors:** G. R. Sridhar, G. Lakshmi

**Affiliations:** ^1^ Department of Endocrinology, Endocrine and Diabetes Centre, Visakhapatnam, India; ^2^ Department of Medicine, Gayatri Vidya Parishad Institute of Health Care and Medical Technology, Visakhapatnam, India

**Keywords:** diagnosis, clinical, hyperinsulinemia, meta analysis, inflammation

## Introduction

The twin epidemics of diabetes and depression occur together, sharing a bidirectional relationship. There is sufficient evidence about their epidemiological, clinical, and pathogenic associations. The two must be managed together rather than in silos (1).

The molecular causes of depression are poorly understood. A complex interaction of many interrelated pathways may operate. Among the suspects are the monoamine system, stress, neurotransmitters, neurotrophins, impaired mitochondrial function, genetics, epigenetics, neuroinflammation, and the gut–brain axis (2). Hyperinsulinemia, which is common to both diabetes and depression, promotes adiposity; as a chronic low-grade inflammatory state, it can impair the hypothalamic–pituitary–adrenal axis, leading to hypercortisolemia and hyperglycemia (3). Immune-related inflammation observed in depression could adversely impact treatment response: therefore, addressing inflammation helps both depression and diabetes (4). It is likely that different factors can trigger the inter-related brain structures and chemical mediators (5). Environmental triggers likely operate *via* epigenetic changes through miRNAs and histone modifications (6).

Polygenic risk scores predict the diagnosis and therapeutic outcome of both diabetes and depression (7). Changes in salivary biomarkers such as cortisol and melatonin in diabetes have the potential to identify depression (8). Anxiety and depression often co-exist in diabetes, with the two having differing effects on glycemic control (9). Recent evidence has shown that management of depressive symptoms can improve anxiety as well (10).

## Highlights of manuscripts in the Research Topic

In this Research Topic, 29 authors from five countries contributed three original articles and one systemic review and network meta-analysis on the diagnosis, etiology, biomarkers, and clinical association of depression and diabetes mellitus.

In contrast to the diagnosis of diabetes mellitus, which is based on objective criteria, the diagnosis of depression is based on subjective assessment by the health care worker. Duong et al. showed that there is a difference in the sensitivities of various criteria to diagnose diabetes, viz., glycosylated hemoglobin (HbA^1^c), fasting plasma glucose (FPG), and HbA^1^c/FPG. From a search of PubMed, Embase, Cochrane Library, and Scopus, 75 studies were included. A bivariate regression model using the Bayesian framework showed that FPG=>123/dl was the best diagnostic test for diabetes mellitus. Clinicians would be helped by the evidence of performance among the different tests to diagnose diabetes mellitus.

Mezuk et al. employed a genetically leveraged study to clarify the relationship between depression and type 2 diabetes, which are believed to operate through psychological, behavioral, and biological processes. The authors drew upon a cohort from the Mood and Immune Regulation in Twins Study to assess social, metabolic, and immune functions in a six-month longitudinal study. They concluded that twin studies have the potential to clarify the biopsychosocial processes underlying the two conditions, and that gene expression studies could further clarify the association. Although the current study did not give a clear answer, it provides direction for assessing pathological disturbances.

Kwon et al. assessed whether the risk of depression and anxiety increases in people with type 2 diabetes in the presence of immune-mediated inflammatory diseases (IMIDs). Considering the potential immune dysregulation in both diabetes and depression, the authors assessed whether the addition of IMIDs in diabetes increased the risk of depression and anxiety. Data were extracted from the nationwide health check-up data from the Korean National Health Insurance Service. A large cohort (n:1,612,705) of subjects without diabetes was followed up for a mean period of 6.4 years. The presence of IMIDs was related to a greater risk of depression and anxiety, with a trend of the association binge greater among those with two or more IMIDs.

Finally, Hargittay et al. provided evidence from Hungary on the effect of depression and anxiety on glycemic control. They reaffirmed that among 338 consecutive subjects with type 2 diabetes mellitus in six primary care offices, anxiety symptoms were more common than depressive symptoms; the latter were associated with poorer glycemic control.

## Conclusion

These four articles both provide clinically relevant guidelines for the management of depression in diabetes and suggest paths to study the association, thereby suggesting ways for their prevention and management.

## Author contributions

All authors listed have made a substantial, direct and intellectual contribution to the work and approved it for publication.
